# MICA-specific nanobodies for diagnosis and immunotherapy of MICA^+^ tumors

**DOI:** 10.3389/fimmu.2024.1368586

**Published:** 2024-03-14

**Authors:** Elisha R. Verhaar, Anouk Knoflook, Novalia Pishesha, Xin Liu, Willemijn J. C. van Keizerswaard, Kai W. Wucherpfennig, Hidde L. Ploegh

**Affiliations:** ^1^ Boston Children’s Hospital, Harvard Medical School, Boston, MA, United States; ^2^ Department of Cell and Chemical Biology, Leiden University Medical Centre, Leiden, Netherlands; ^3^ Division of Immunology, Boston Children’s Hospital, Boston, MA, United States; ^4^ Department of Pediatrics, Harvard Medical School, Boston, MA, United States; ^5^ Department of Cancer Immunology and Virology, Dana-Farber Cancer Institute, Boston, MA, United States

**Keywords:** MICA, NKG2D, NKG2D ligands, cancer, nanobodies, VHHs, immuno-oncology, nanobody drug conjugate

## Abstract

MICA and MICB are Class I MHC-related glycoproteins that are upregulated on the surface of cells in response to stress, for instance due to infection or malignant transformation. MICA/B are ligands for NKG2D, an activating receptor on NK cells, CD8^+^ T cells, and γδ T cells. Upon engagement of MICA/B with NKG2D, these cytotoxic cells eradicate MICA/B-positive targets. MICA is frequently overexpressed on the surface of cancer cells of epithelial and hematopoietic origin. Here, we created nanobodies that recognize MICA. Nanobodies, or VHHs, are the recombinantly expressed variable regions of camelid heavy chain-only immunoglobulins. They retain the capacity of antigen recognition but are characterized by their stability and ease of production. The nanobodies described here detect surface-disposed MICA on cancer cells *in vitro* by flow cytometry and can be used therapeutically as nanobody-drug conjugates when fused to the Maytansine derivative DM1. The nanobody-DM1 conjugate selectively kills MICA positive tumor cells *in vitro.*

## 1 Introduction

The Class I MHC-like molecules MICA and MICB are stress-induced surface glycoproteins, absent from healthy cells but upregulated on virus-infected or malignantly transformed human cells (1). MICA/B are ligands for NKG2D, an activating receptor on NK cells, CD8^+^ T cells, and γδ T cells (2). Upon engagement of NKG2D, these cytotoxic cells can eradicate MICA-positive targets, assisted by secretion of cytokines (3–5). Elevated levels of MICA/B occur in hematopoietic malignancies, as well as in epithelial solid tumors such as colorectal cancer (6), ovarian cancer (7), cervical cancer (8), breast cancer (9), pancreatic cancer (10), melanoma (11) and cholangiocarcinoma (12). MICA/B are thus considered possible targets for immunotherapy.

Nanobodies, a registered trademark, are also referred to as VHHs. They are the smallest immunoglobulin fragments that retain the capacity of antigen binding. They are the recombinantly expressed variable regions of camelid heavy chain-only immunoglobulins (13). Nanobodies have a short circulatory half-life, are poorly immunogenic, and show excellent tissue penetration compared to conventional full-sized immunoglobulins (14, 15). Many nanobodies do not require disulfide bonds for their stability, nor do they depend on glycosylation for expression. They are therefore easily and affordably produced in prokaryotic cells (16–18). Nanobodies have proven valuable as the point of departure for the construction of PET imaging agents (19–24), nanobody-drug conjugates (25–27), and chimeric antigen receptors in cell-based therapies (28–38).

Because MICA is expressed on stressed and cancerous cells, the ability to detect such aberrations *in vivo* would be an important diagnostic tool to detect premalignant and malignant lesions. Here, we report the generation of nanobodies that recognize MICA, and apply these nanobodies to detect surface-bound MICA *in vitro* by flow cytometry. Fused to the microtubule inhibitor Maytansine (DM1), these nanobodies can be used therapeutically as nanobody-drug conjugates.

## 2 Materials and methods

### 2.1 Alpaca immunization and phage library construction

We immunized an alpaca with 250 ug of the purified extracellular portion of MICA*009 (obtained by baculovirus expression in the lab of K.W. Wucherpfennig (39)) comprising the α1, α2, and α3 domains in alum adjuvant, followed by 3 booster injections at 2-week intervals. Immunizations were carried out by Camelid Immunogenics. The immune response of the animal was checked by immunoblot (**Supplementary Figure 1**). Briefly, 1 μg of antigen was resolved by SDS PAGE and transferred to a PVDF membrane. The membrane was incubated with at 1:5000 dilution of alpaca serum collected 2 weeks after the last boost. HRP-linked goat-anti-llama (0.05 μg/mL; Bethyl, NC9656984) was used as the secondary antibody. Membranes were developed with ECL Western Lightning Plus. Mononuclear cells from peripheral blood of the immunized alpaca were isolated by Ficoll gradient separation. The VHH library was generated according to an established protocol (Maas et al., 2007). Briefly, RNA was extracted (RNeasy RNA purification kit, Qiagen) and cDNA was prepared (Superscript III first-strand synthesis system, Invitrogen). The DNA sequences from conventional and heavy-chain only Ig genes are not distinguishable based on the use of specific primers, but two distinct hinge regions are generated between the VHH domain and the CH2 region. We amplified the VHH repertoire from the alpaca using VHH-specific primers that target these hinge sequences (**Supplementary Table 1**). We pooled the VHH PCR products and ligated them into a phagemid vector in-frame with the pIII gene of the M13 phagemid to construct a phagemid library display. We performed two rounds of panning against MICA*009 immobilized on an ELISA plate, following previously described protocols (40).

### 2.2 Production of recombinant VHHs and sortase reactions

DNA from positive clones was sequenced and 9 clones were selected for further characterization. The relevant VHH sequences were subcloned into a pHEN6 expression vector with C-terminal modifications, so that each nanobody sequence included an LPETG motif recognized by sortase A, followed by a (His)_6_-tag to facilitate recovery and purification. Briefly, VHH sequences were amplified from the phagemid vector by PCR (primers in **Supplementary Table 1**) and the pHEN6 vector was linearized using the NcoI and BstEII restriction enzymes. Gibson assembly was performed following manufacturer’s directions (Gibson Assembly^®^ Master Mix, NEB). Positive VHH clones were expressed in WK6 *E.Coli* in terrific broth and periplasmic protein expression was activated by induction with isopropyl β-D-thiogalactopyranoside (1 mM) at an OD600 of 0.6. VHHs were harvested from the periplasm by osmotic shock. The C-terminal (His)_6_-tag allows purification of the recombinant proteins using Ni-NTA agarose beads (Qiagen), followed by FPLC purification on an S75 column by FPLC (ÄKTA, Cytiva Life Sciences). Sortase reactions were performed by incubating each nanobody with a 10-fold molar excess of GGG-nucleophile in the presence of 25 µM Sortase 7M (41) overnight at 4°C. Because the LPETG sequence is cleaved during transpeptidation, the (His)_6_-tag immediately C-terminal of the LPETG motif is lost. This allows enrichment of the desired modified product by depletion of His-tagged sortase and unreacted nanobody on a NiNTA matrix, while the unbound fraction contains the modified nanobody.

### 2.3 Competitive ELISA and estimation of binding affinity

An ELISA was performed to determine the concentration at which each biotinylated nanobody showed ~80% binding to recombinant MICA*009 (5 mg/mL) immobilized on an ELISA plate. Biotinylated nanobody at a concentration that yielded 80% of the maximum attainable binding value was then mixed with a 500-fold excess of unlabeled competitor nanobody and allowed to compete for binding to 5 μg/mL MICA*009 coated on an ELISA plate. Plates were incubated with streptavidin-HRP (0.00025 μg/mL) for 45-60 minutes at room temperature. After addition of TMB substrate, absorbance was read out at 450 nm on a Spectramax iD5 plate reader (Molecular Devices). If the unlabeled nanobody binds to an epitope distinct from that recognized by the biotinylated nanobody, no diminution of the signal at 450 nm is expected. Nanobodies that recognize the same epitope as that seen by the biotinylated nanobody will show a reduction in the signal at 450nm.

We estimated the binding affinity of VHH-A1 and VHH-H3 by performing an ELISA as previously described (42). Briefly, we incubated plates coated with 100μL PBS containing 2.5 μg/mL recombinant MICA*009 or GFP as negative control with biotinylated VHH-A1 and VHH-H3 in various concentrations (10-fold serial dilutions; 0.000001 nM – 1000 nM). Streptavidin-HRP at 0.00025 μg/mL was used as detection agent. After addition of TMB substrate, absorbance was read at 450 nm on a Spectramax iD5 plate reader (Molecular Devices). Binding affinity was estimated by calculating the IC50 obtained from three experimental replicates with each sample added in duplicates. Recombinant MICA*009 was produced by transfection of EXPI-293 cells with pcDNA3.1(+) vector encoding for extracellular MICA*009 containing a C-terminal LPETG sortase motif followed by a His (6)-tag to facilitate recovery and purification on a NiNTA matrix (**Supplementary Figure 2**). EXPI-293 cells were transfected using the ExpiFectamine™ 293 Transfection Kit, according to manufacturer’s directions (Gibco).

### 2.4 Cell culture

B16F10 and EL-4 cells and their MICA^+^ transfectants were a gift from the lab of Kai Wucherpfennig. B16F10 cells were cultured in complete DMEM (DMEM with 4.5 g/L glucose, substituted with 10% Fetal Bovine Serum (FBS) and 100 U/mL penicillin/streptomycin). EL-4 cells were cultured in complete RPMI 1640 (RPMI 1640, substituted with 10% FBS and 100 U/mL penicillin/streptomycin). Cells were maintained at optimal densities in a humidified 5% CO2 incubator at 37°C.

### 2.5 Flow cytometry

EL-4 WT and MICA^+^ cells, or B16F10 WT and MICA^+^ cells, were stained with biotinylated VHH-A1 and VHH-H3 for 30 minutes on ice, washed, and incubated with a cocktail of Streptavidin-conjugated PE at 0.0025 μg/mL (Invitrogen) and 2 μg/mL propidium iodide (Life technologies) for EL-4 or LIVE/DEAD™ Fixable Violet Dead Cell Stain Kit (Invitrogen) for B16F10, both according to manufacturer’s directions for 30 minutes on ice. Cells were analyzed on an LSR Fortessa flow cytometer (BD Biosciences). Gating strategies were based on cell lines stained with the appropriate controls, where single cells and live cells were appropriately selected.

### 2.6 VHH-drug conjugate creation and *in vitro* cytotoxicity assays

VHH-DM1 was produced in a sortase-mediated transpeptidation reaction. Briefly, 500-1000 μg of VHH containing a C-terminal LPETG-motif was mixed with a 10-fold molar excess of GGG-DM1 and incubated with 25 μM Sortase for 16 hours at 4°C. GGG-DM1 was produced in-house by modifying a GGG-peptide linker to contain a maleimide group and allowing it to react with the thiol group on DM1 (Broadpharm) (**Supplementary Figure 3A**). Unreacted VHH and Sortase, both containing a (His)_6_-tag, were depleted by incubation with NiNTA agarose (Qiagen or Prometheus). Excess free GGG-DM1 was removed by desalting on a PD-10 desalting column (Cytiva). We plated 4000 cells/well in a 96-well plate and incubated cells with serial 3-fold dilutions of VHH-drug adduct or free DM4 (Broadpharm), a structural analog of DM1 (**Supplementary Figure 3B**) at 37°C in a humidified 5% CO2 atmosphere. After 72 hours, we measured cell viability by CellTiter GloTM assay according to the manufacturer’s directions (Promega). For co-culture experiments, MICA expression was determined after a 72-hour incubation. Each treatment was performed in duplicate. For flow cytometry, the duplicate wells of each condition were combined, and the cell mixture was stained with 0.0006 μg/mL biotinylated anti-human MICA/B antibody (Clone 6D4, Biolegend) for 30 minutes on ice. Cells were washed and incubated with Streptavidin-conjugated PE at 0.0025 μg/mL (Invitrogen) and LIVE/DEAD™ Fixable Violet Dead Cell Stain Kit according to manufacturer’s directions (Invitrogen) for 30 minutes on ice. Cells were washed and viability and MICA positivity were determined by flow cytometry on an LSR Fortessa flow cytometer (BD Biosciences).

### 2.7 Statistical analysis

All statistical analysis was performed with GraphPad Prism 8. Flow cytometry data was analyzed with FlowJo (v10.8.1 and v10.9.0).

## 3 Results

### 3.1 Alpaca immunization and phage display panning yields MICA-specific nanobodies

**Figure 1:**
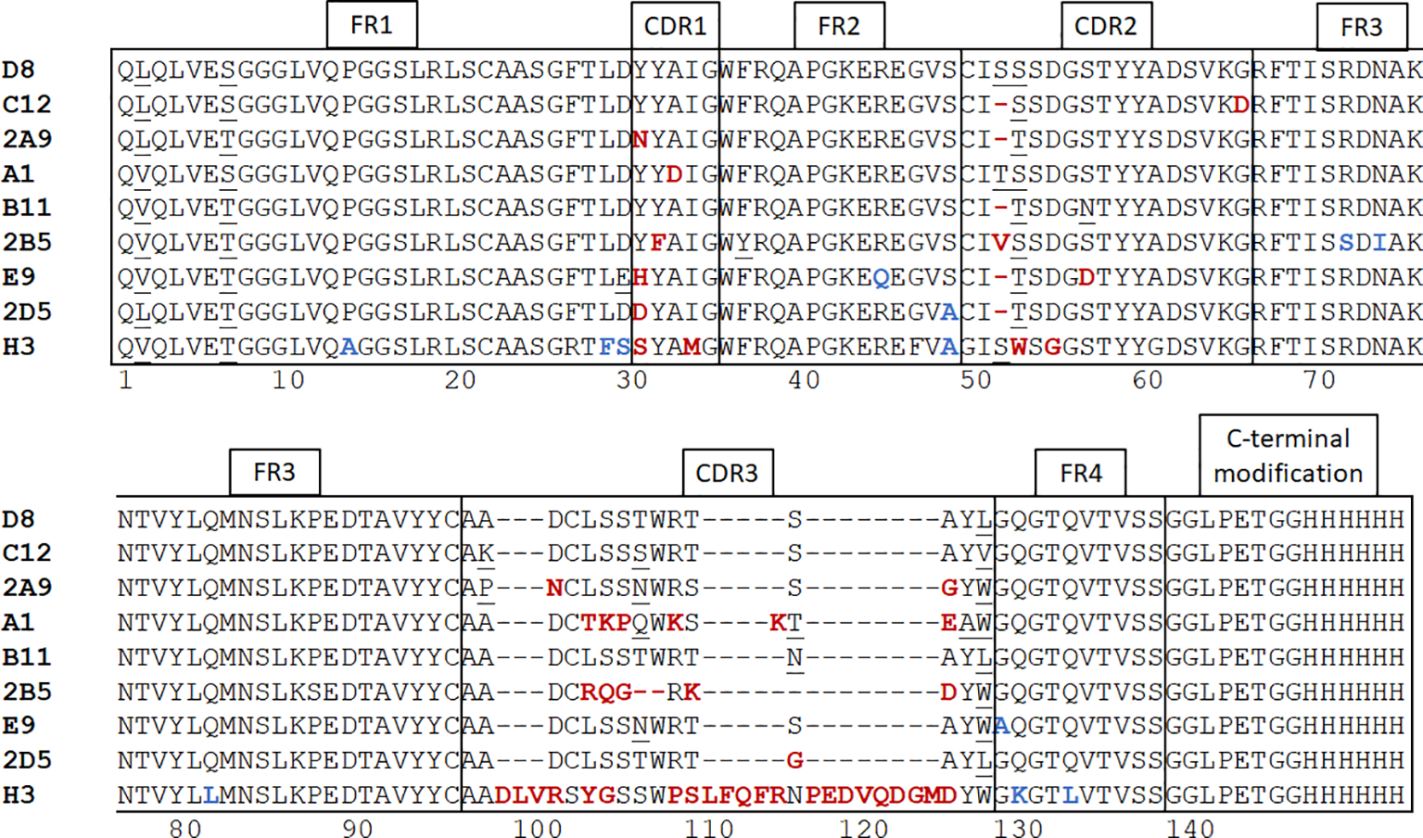
Alpaca immunization and nanobody panning. After construction of a phage display library and screening for positive clones with plate-based panning, nanobody sequences were determined and 9 unique clones were selected. Neutral amino acid substitutions attributable to somatic hypermutations are underscored. Unique substitutions in framework regions are highlighted in blue and in CDR’s are highlighted in red. Nanobodies harboring such mutations are more likely derived from different germline V regions rather than somatic hypermutation. The framework regions of nanobodies D8 and C12 are identical. The alpaca IGHHV-3-3*01 gene is the possible germline version of these nanobodies (44). The single difference of VHH A1 with D8 and C12 in its framework regions is an L2V substitution. A1 may thus be derived from the same germline V gene as D8 and C12 by a single (somatic) point mutation. The framework regions of nanobodies 2A9 and 2D5 are mostly identical to each other, with a single S49A substitution between them. Nanobody E9 has both a D29E and a R45Q substitution, indicating that E9 may be derived from a different V gene. In comparison with the other MICA-specific nanobodies, H3 has the largest number of differences in its framework regions and is clearly derived from a different germline V gene, likely the alpaca IGHHV3-1*01 (44). The CDR1 and CDR2 regions are mostly conserved. The most obvious deviation is a deletion at position 53 in VHH C12, B11, 2A9, 2D5, and E9. The MICA-specific nanobodies have CDR3 regions of 13-16 amino acids, but H3 has a 31-residue CDR3. Except for VHH H3, A1 and 2B5, the remaining CDR3 regions are enriched for the sequence “AxDCLSSxWRx”. The VHH sequences were subcloned into the pHen6 expression vector and modified at the C-terminus to contain an LPETG motif and (His)_6_ tag.

We immunized an alpaca with purified recombinant MICA*009 in alum adjuvant, followed by 3 booster injections at 2-week intervals. We checked the immune response of the animal by immunoblot using serum samples collected prior to each boost. Having recorded a positive response after the 3rd boost, construction of a phage display library, followed by screening for MICA-reactive hits, yielded positive clones. DNA from positive clones was sequenced and 9 clones were selected for further characterization. Because nanobodies interact with their antigen mainly via their CDR3 region, and to a lesser extent via the germline-encoded CDR1 and CDR2 (43), we chose clones that were unique in their CDR3. A detailed comparison of the nanobody clones based on sequence similarity in the framework and CDR regions is described in the caption of **Figure 1**.

Relevant VHH sequences were subcloned into a pHEN6 expression vector to encode a VHH product with C-terminal modifications, so that each VHH sequence included an LPETG motif at its C-terminus, recognized by sortase A, and a (His)_6_-tag to facilitate recovery and purification (**Figure 1**). This arrangement enables the installation of fluorophores, biotin, and other substituents by a site-specific and efficient sortase-catalyzed transpeptidation reaction (41). Because the LPETG sequence is cleaved during transpeptidation, the (His)_6_-tag immediately C-terminal of the LPETG motif is lost. This allows enrichment of the desired modified product by depletion of His-tagged sortase and unreacted nanobody on a NiNTA matrix, while the unbound fraction contains the modified nanobody.

### 3.2 Nanobodies recognize recombinant MICA and surface-exposed MICA on cancer cells

**Figure 2:**
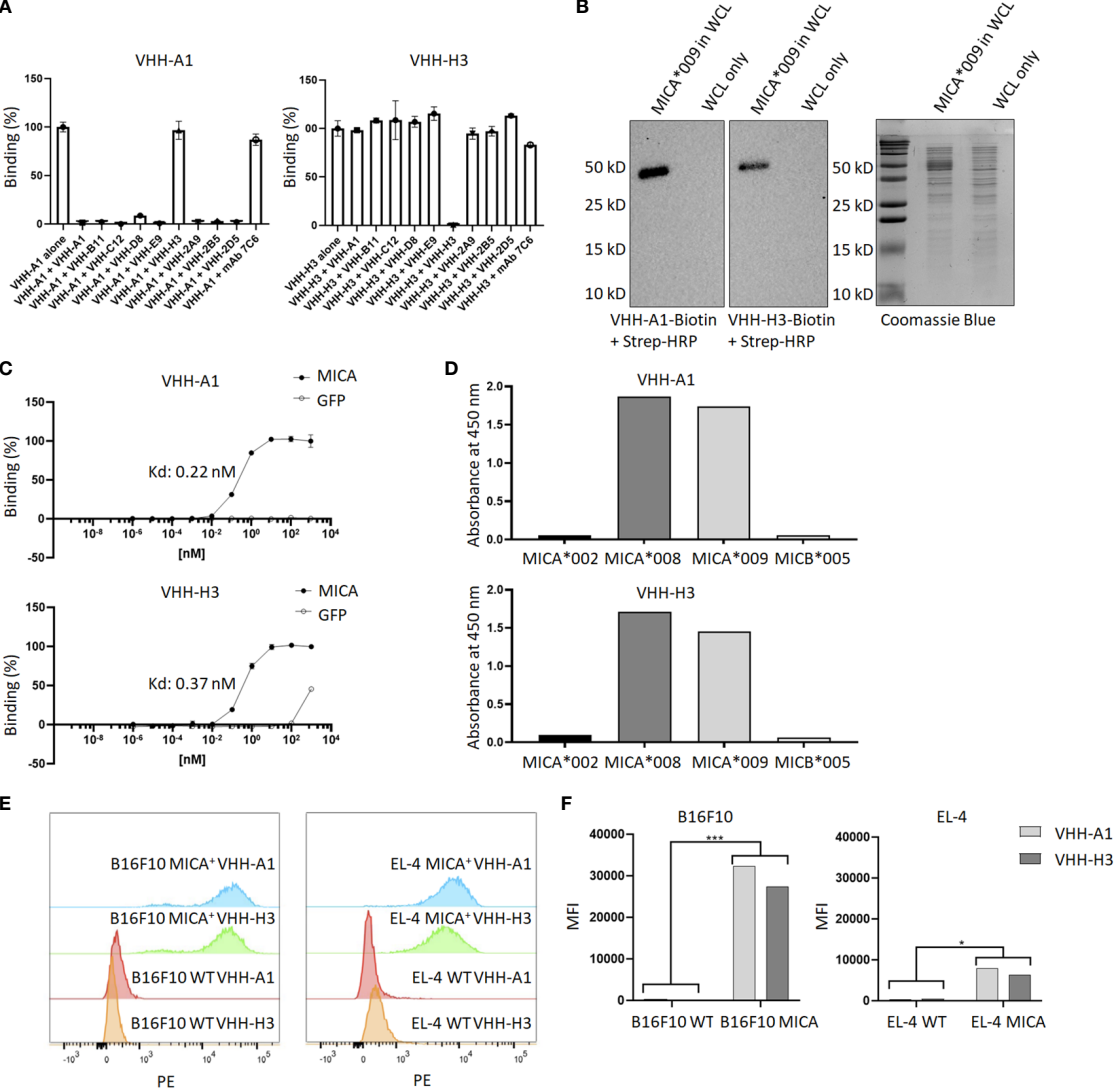
Characterization of MICA-specific VHHs. **(A)** Cross-competition ELISA shows that VHH-A1 and VHH-H3 recognize distinct epitopes on MICA. Neither VHH cross-competes for binding with the monoclonal antibody 7C6. **(B)** VHH-A1 and VHH-H3 recognize MICA in immunoblot. 500 ng recombinant MICA*009 in non-specific *E. coli* whole cell lysate (WCL) was separated by SDS-PAGE and transferred to a PVDF membrane. Blots were stained with 1 μg/mL biotinylated VHH-A1 or VHH-H3 respectively. Detection with strep-HRP (0.3 ng/mL) shows a clear signal for both VHHs. **(C)** Binding affinity as estimated by ELISA coated with 2.5 μg/mL recombinant MICA*009, or GFP as the negative control. Estimated Kd values are 0.22 nM and 0.37 nM for VHH-A1 and VHH-H3 respectively. **(D)** ELISA coated with different recombinant MICA alleles shows that VHH-A1 and VHH-H3 both recognize MICA*008 and MICA*009. **(E)** Flow cytometry with biotinylated VHH-A1 and VHH-H3, using streptavidin-conjugated PE as secondary agent, shows a clear signal in the PE channel for MICA^+^ EL-4 and B16F10 cells, but not for the WT cells, indicating recognition of membrane-disposed MICA on the surface of cells by both nanobodies. Gating strategies for flow cytometry are shown in **Supplementary Figure 4**. **(F)** We calculated the MFI after flow cytometry. The MFI of B16F10 WT cells was 394 for VHH-A1 and 299 for VHH-H3. The MFI of B16F10 MICA^+^ cells was 23430 for VHH-A1 and 27411 for VHH-H3. The MFI of EL-4 WT was 310 for VHH-A1 and 511 for VHH-H3. MFI of EL-4 MICA^+^ cells was 7955 for VHH-A1 and 6417 for VHH-H3. We averaged the MFI from the WT or MICA^+^ cells and determined a significant difference in nanobody staining of WT versus MICA^+^ cells (p = 0.00713 for B16F10; p = 0.0128 for EL-4, calculated by multiple T-test).

To determine whether the isolated MICA-specific nanobodies recognized similar or distinct epitopes on MICA, we performed cross-competition experiments by ELISA. Competition of unlabeled nanobodies with a biotinylated nanobody for binding to MICA showed that this set of nanobodies recognizes two distinct epitopes, one defined by the H3 nanobody and the second by all the other nanobodies. None of the nanobodies compete for binding with the 7C6 monoclonal antibody, an agent that inhibits shedding of MICA (45) (**Figure 2A**). Typically, not all nanobodies are suitable for use in immunoblotting experiments, but the biotinylated versions of A1 and H3 yielded a strong and specific signal in immunoblots on recombinant MICA (**Figure 2B**). The binding affinities of VHH-A1 and VHH-H3 are both in the nanomolar range, at ~0.2 and ~0.4 nM for A1 and H3 respectively (**Figure 2C**), as estimated by ELISA assay. By examining the binding of the A1 and H3 nanobodies to a subset of MICA/B allelic products, available in purified form, we conclude that the A1 and H3 nanobodies recognize the MICA*008 and MICA*009 alleles (**Figure 2D**) which, combined, cover 51.1% of the Caucasian population (46). To verify that A1 and H3 also recognize surface-disposed MICA, we used B16F10 transfectants that express MICA*009, and EL-4 transfectants that express MICA*008, with B16F10 and EL-4 wild type cells serving as negative controls. Both A1 and H3 showed excellent staining of the MICA transfectants by flow cytometry and yielded no signal for the untransfected parental cell lines (**Figure 2E**) with a significant difference determined by mean fluorescence intensity (MFI) (**Figure 2F**). Gating strategies are shown in **Supplementary Figure 4**.

### 3.3 Anti-MICA nanobodies fused to Maytansine (DM1) for targeted cytotoxicity of MICA^+^ cancer cells

**Figure 3:**
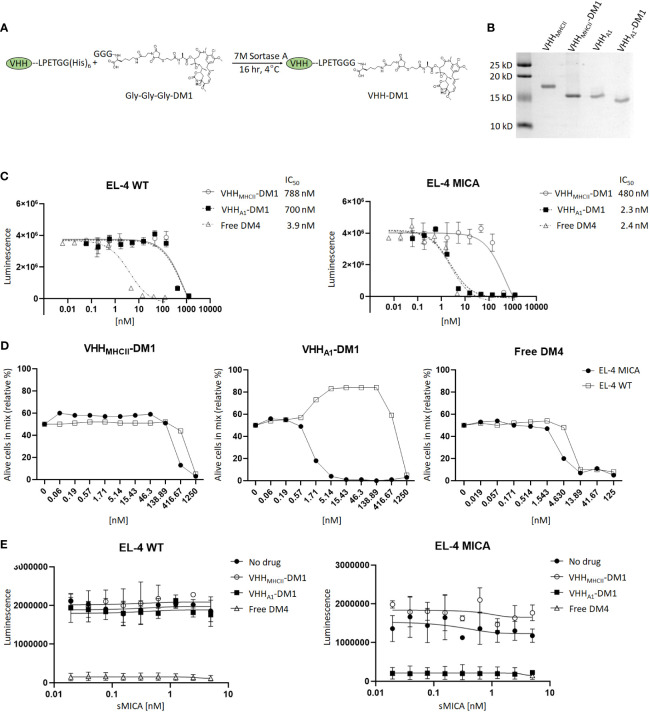
Anti-MICA VHHs as nanobody-drug conjugate with the Maytansine derivative DM1. **(A)** We ligated the microtubule inhibitor Maytansine GGG-DM1 to VHH-A1 or VHH_MHC-II_ as non-targeting control through sortase-mediated transpeptidase reaction. **(B)** Because GGG-DM1 has a slight positive charge, the modified VHHs will migrate slightly lower on the SDS-PAGE gel compared to the unmodified VHHs. **(C)** The *in vitro* cytotoxicity assay was performed with limited dilutions of VHH_MHC-II_-DM1, VHH_A1_-DM1, or free DM4 on EL-4 WT cells and their MICA^+^ counterparts. After incubation for 72 hours, we measured cell viability by CellTiter Glo™ assay. MICA^+^ cells treated with VHH_A1_-DM1 showed a significant reduction in IC_50_, and thus a reduction in viability with smaller amounts of drug added, compared to similarly treated WT cells, or cells treated with the non-targeting VHH_MHCII_-DM1. **(D)** We co-cultured EL-4 WT and EL-4 MICA^+^ cells at a 1:1 ratio and added VHH_MHCII_-DM1, VHH_A1_-DM1, or free DM4 at different concentrations. Viability of EL-4 WT and MICA^+^ cells was determined using a live/dead cell stain. MICA^+^ cells were stained with a biotinylated anti-MICA mAb, using streptavidin-PE as secondary agent. Gating on live cells and PE showed elimination of MICA^+^ cells at VHH-drug adduct concentrations between 1.71 nM and 416 nM for VHH_A1_-DM1. A difference in [WT : MICA] was not observed in cells treated with VHH_MHCII_-DM1 or free DM4. Gating strategies for flow cytometry are shown in **Supplementary Figure 5**. **(E)** We incubated EL-4 WT and MICA^+^ cells with 2.5 nM of VHH_MHCII_-DM1, VHH_A1_-DM1, or free DM4 in the presence of sMICA (two-fold dilutions; 0-5 nM/0-170 ng/mL) for 72 hours. We measured viability by CellTiter Glo™ assay. We did not observe a decreased effect on cytotoxicity of VHHA1-DM1 on MICA^+^ cells with addition of sMICA in the medium.

The reactivity of VHH-A1 and VHH-H3 make them appealing candidates for the construction of nanobody-drug conjugates. To test this, we ligated the Maytansine derivative DM1, a microtubule disrupting agent, to VHH-A1 or to a VHH that targets mouse MHC-II (VHH_MHC-II_) (47) as a negative control via a sortase-mediated transpeptidation reaction (**Figure 3A**) and confirmed successful ligation with SDS-PAGE (**Figure 3B**). We performed an *in vitro* cytotoxicity assay by titration of VHH_MHC-II_-DM1, VHH_A1_-DM1, or free DM4 (a functional analog of DM1) on EL-4 WT and MICA^+^ cells. EL-4 MICA^+^ cells were sensitive to VHH_A1_-DM1, with a stronger cytotoxic effect at lower doses of the VHH-drug conjugate compared to VHH_MHC-II_-DM1, as estimated by IC50. The IC50 of VHH_A1_-DM1 treated EL-4 MICA^+^ cells was comparable to that of cells treated with free DM4. Similarly treated WT cells showed no obvious reduction in viability with either nanobody-drug conjugate (**Figure 3C**).

To further validate selectivity of VHH_A1_-DM1 for MICA^+^ cells, we co-cultured EL-4 WT and EL-4 MICA^+^ cells at a 1:1 ratio, and added VHH_MHCII_-DM1, VHH_A1_-DM1, or free DM4 at different concentrations. We determined the ratio of viable EL-4 WT and EL-4 MICA^+^ cells after 72 hours by flow cytometry using a live/dead cell stain. We stained the MICA^+^ cells in the co-culture with a biotinylated αMICA mAb, using streptavidin-conjugated PE as secondary reagent. Gating on live cells and MICA^+^ cells showed specific elimination of MICA^+^ cells at adduct concentrations between 1.71 nM and 416 nM for VHH_A1_-DM1. A difference in ratio between WT and MICA^+^ cells was not observed in cells treated with VHH_MHCII_-DM1 or free DM4. Because WT cells proliferate slightly faster than MICA^+^ cells in culture, the distribution shifted to ~65% WT and 35% MICA^+^ cells after 72 hours in culture. Thus, numbers were normalized according to the percentage of cells of either line in the untreated (“0 nM”) group (**Figure 3D**). Gating strategies are shown in **Supplementary Figure 5**.

Tumor cells can downregulate surface expression of MICA through shedding, mediated by proteolytic cleavage at the α3 domain. Increased levels of soluble MICA (sMICA) in the serum of patients are associated with poor prognosis and worse disease progression (10, 48–50). To address the possible competition of sMICA for binding with the anti-MICA nanobody, we performed an *in vitro* cytotoxicity assay. EL-4 WT and MICA^+^ cells were incubated with VHH_MHCII_-DM1, VHH_A1_-DM1, or free DM4 at a fixed concentration of 2.5 nM, in the presence of sMICA at various concentrations (serial 2-fold dilutions; 0-5 nM/0-170 ng/mL). We observed no reduction in cytotoxicity of VHH_A1_-DM1 on MICA^+^ cells upon addition of sMICA to the medium (**Figure 3E**). Publications report concentrations of sMICA in the serum of MICA^+^ patients in the range of 0.1-15 ng/mL (51–53) which is at least 10-fold lower than the sMICA concentration in our competition assay. We thus expect little to no impact of sMICA in patients’ serum on the ability of these nanobodies to target membrane-bound MICA *in vivo*.

## 4 Discussion

MICA and MICB are Class I MHC-related proteins expressed on stressed and cancerous cells. Their presence can serve not only as a diagnostic marker but may also be exploited as a target for therapy. While the typical immunoglobulins exert their functional properties through Fc effector functions, their size compromises efficient tissue penetration. Nanobodies offer an appealing alternative to immunoglobulins for the purpose of launching an immune attack on MICA-positive tumors. Nanobodies are characterized by their small size, showing superior tissue penetration compared to intact immunoglobulins, and ease of production and modification (14, 15, 17, 18). Lastly, nanobodies are poorly immunogenic, presumably because of their considerable sequence homology with human V_H_ regions (44). Because nanobodies lack an Fc portion, for them to exert cytotoxic activity they require functionalization, for example with a cytotoxic drug creating a nanobody-drug conjugate, as done here for the VHH-DM1 adducts. Compared to antibody-drug conjugates using conventional immunoglobulins, the small size of the nanobody allows superior penetration into tumor tissue. Furthermore, owing to the relatively short circulatory half-life, the nanobody-drug conjugate that is not bound to its target will be eliminated more quickly from the circulation, resulting in less systemic cytotoxicity by slow release of the drug attached to the antibody-drug conjugate.

We produced and characterized in further detail two nanobodies, A1 and H3, that recognize the MICA alleles *008 and *009 with nM affinities. An analysis of the MICA-specific nanobodies shows that they are unique sequences, thus the isolated nanobodies were likely derived from a few different germline V genes (see **Figure 1** and legend). The germline sequences of the V genes of the (outbred) alpaca used for immunization are not known. We can only compare the sequences of the MICA-specific nanobodies with each other, and with reference germline sequences from unrelated alpacas.

The alpaca IGHHV-3-3*01 gene is the possible germline version of the D8 and C12 nanobodies (44). The single difference of VHH A1 with D8 and C12 in its framework regions is an L2V substitution, thus A1 may be derived from the same germline V gene as D8 and C12 by somatic mutation. Nanobody E9 has a D29E and an R45Q substitution, indicating that E9 may be derived from a different V gene. In comparison with the other MICA-specific nanobodies, H3 has the largest number of differences in its framework regions and is clearly derived from a different germline V gene, likely the alpaca IGHHV3-1*01 (44).

Highly similar CDR regions, specifically CDR3, imply recognition of related antigens (54–57). For the MICA-specific nanobodies, the CDR1 and CDR2 regions are mostly conserved. The most obvious deviation in the CDR2 region is a deletion at position 53 in VHH C12, B11, 2A9, 2D5, and E9. Somatic hypermutation can produce deletions and insertions in V genes (58–60) but given the overall similarity in framework regions, the use of a distinct V gene that lacks residue 53 is the more plausible explanation. Except for H3, A1 and 2B5, the remaining CDR3 regions are enriched for the sequence “AxDCLSSxWRx”.

We show that these nanobodies bind to surface-disposed MICA on cells and can thus be used for diagnostic and therapeutic purposes. The specific targeting of MICA^+^ cells make them suitable candidates as diagnostic markers, as building blocks for nanobody-drug conjugate, or for the construction of chimeric antigen receptors (29, 30, 37, 61). MICA and MICB are highly polymorphic in the human population, with hundreds of alleles for MICA and MICB identified so far (46, 62). The isolated nanobodies were tested for recognition of the MICA alleles *002, *008 and *009, and MICB allele *005. Of the tested alleles, the nanobodies recognize MICA*008 and MICA*009, which together cover over 50% of the investigated German population (46). Expanding the nanobody pool to cover a larger portion of the alleles of MICA and MICB should be considered. We recognize the limitations of using a MICA^+^ cell line obtained by transfection. The availability of a suitable patient-derived cell line that expresses the correct alleles of MICA is a limiting factor, an issue worth exploring in future research.

We created a nanobody-drug conjugate by conjugating the microtubule inhibitor DM1 to VHH-A1. We show increased cytotoxicity of MICA^+^ tumor cells compared to WT tumor cells *in vitro*, with efficacy comparable to that of free drug but with much higher specificity for MICA^+^ cells. The production of these nanobody adducts should be scaled up for testing on *in vivo* tumor models. The creation of different VHH-drug combinations, for example by inclusion of DNA damaging agents or other cytotoxic drugs (63, 64), or even radiopharmaceuticals for targeted radiotherapy (65, 66), deserves consideration as well.

Cleavage of the α3 domain involving the disulphide isomerase ERp5 and ADAM-type proteases such as ADAM10 and ADAM17 (48–50, 67, 68), and thus shedding of the MICA/B from the cancer cell surface, may lead to immune evasion and failure to be recognized by NKG2D-positive cytotoxic cells. The monoclonal antibody 7C6 inhibits the shedding of MICA/B, and thus increases the density of MICA/B proteins on the surface of tumor cells (45) Although we saw no reduction in efficacy of VHH_A1_-DM1 on MICA^+^ cells upon addition of sMICA to the medium, the combination of anti-MICA nanobody adducts with the 7C6 antibody might therefore be therapeutically more attractive than either treatment alone.

## Data availability statement

The datasets presented in this study can be found in online repositories. The names of the repository/repositories and accession number(s) can be found below: 10.6084/m9.figshare.25289806.

## Ethics statement

The animal study was approved by IACUC University of Massachusetts Amherst. The study was conducted in accordance with the local legislation and institutional requirements.

## Author contributions

EV: Writing – original draft, Visualization, Validation, Supervision, Methodology, Investigation, Formal analysis, Data curation, Conceptualization. AK: Writing – review & editing, Investigation, Data curation. NP: Writing – review & editing, Resources, Investigation. XL: Writing – review & editing, Resources. WvK: Writing – review & editing, Investigation. KW: Writing – review & editing, Resources. HP: Writing – review & editing, Writing – original draft, Supervision, Project administration, Funding acquisition, Conceptualization.
