# Supplementary material for: MICA-specific nanobodies for diagnosis and immunotherapy of MICA+ tumors
Source: Front Immunol. 2024 Mar 14;15:1368586. doi: 10.3389/fimmu.2024.1368586 (PMC10973119; doi:10.3389/fimmu.2024.1368586)
Supplement: Supplementary file 1 [file DataSheet_1.docx]

**Supplementary figures**

**
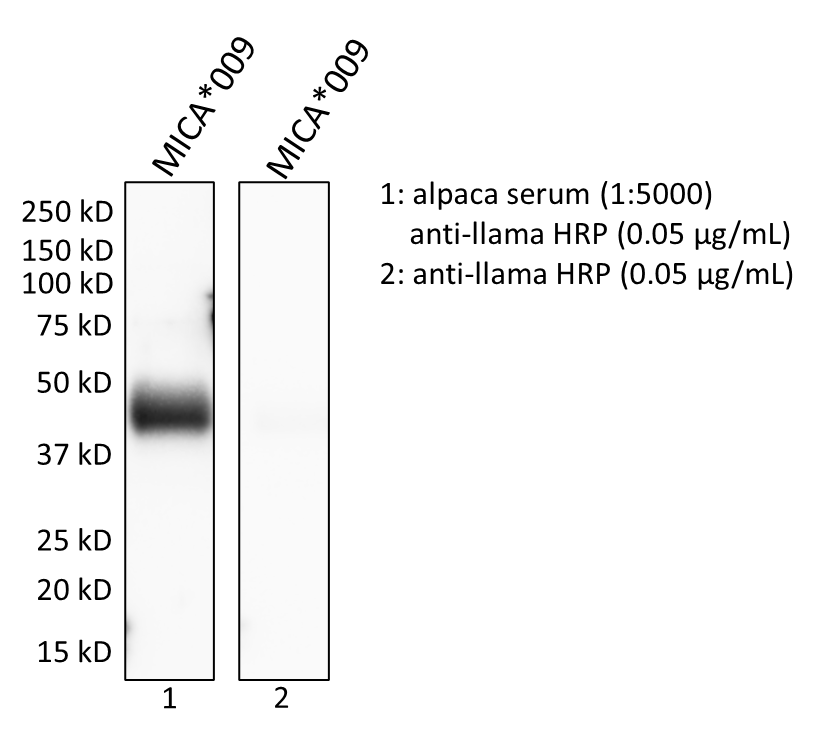
**

**Supplementary figure 1.** **Immunoblot to determine the immune response of the alpaca after 4 immunizations with recombinant MICA*009.** 1 μg of antigen was resolved by SDS PAGE and transferred to a PVDF membrane. The membrane was incubated with at 1:5000 dilution of alpaca serum collected 2 weeks after the last boost. HRP-linked goat-anti-llama (0.05 μg/mL; Bethyl, NC9656984) was used as the secondary antibody. Membranes were developed with ECL Western Lightning Plus. To rule out a non-specific signal from the secondary antibody, a membrane with MICA*009 was incubated with the secondary antibody only and developed under the same conditions.


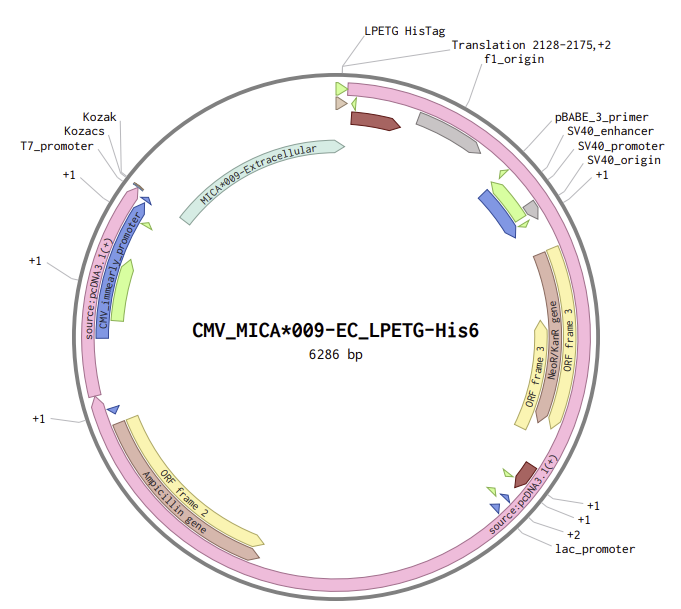
**Supplementary figure 2. pcDNA3.1(+) vector containing the sequence for extracellular, secreted MICA*009-LPETG-His(6).**

**
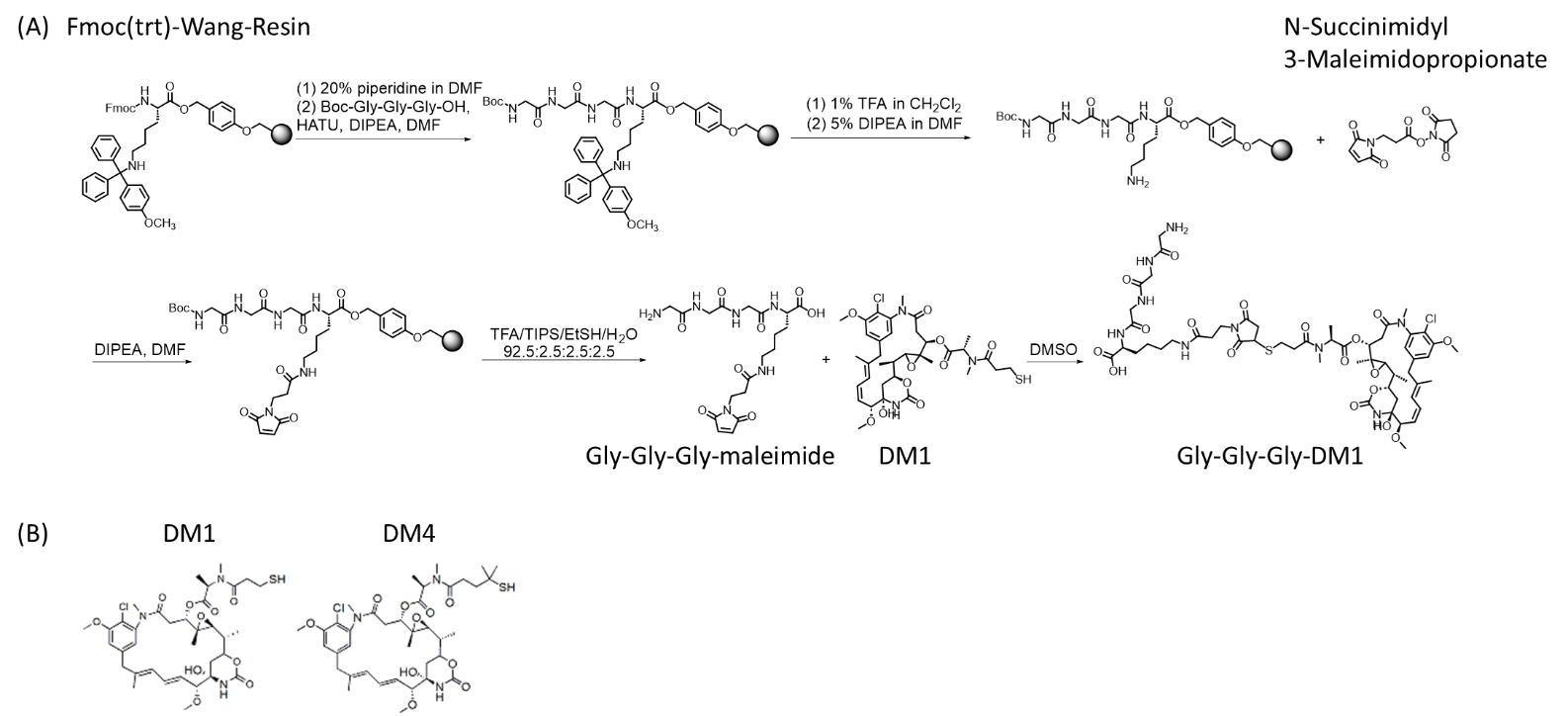
Supplementary figure 3. Chemical synthesis of GGG-DM1.** (A) We modified a GGG-peptide linker to contain a maleimide group and allowed it to react with the thiol group of commercially obtained DM1. (B) Chemical structures of unmodified DM1 and DM4.

# **
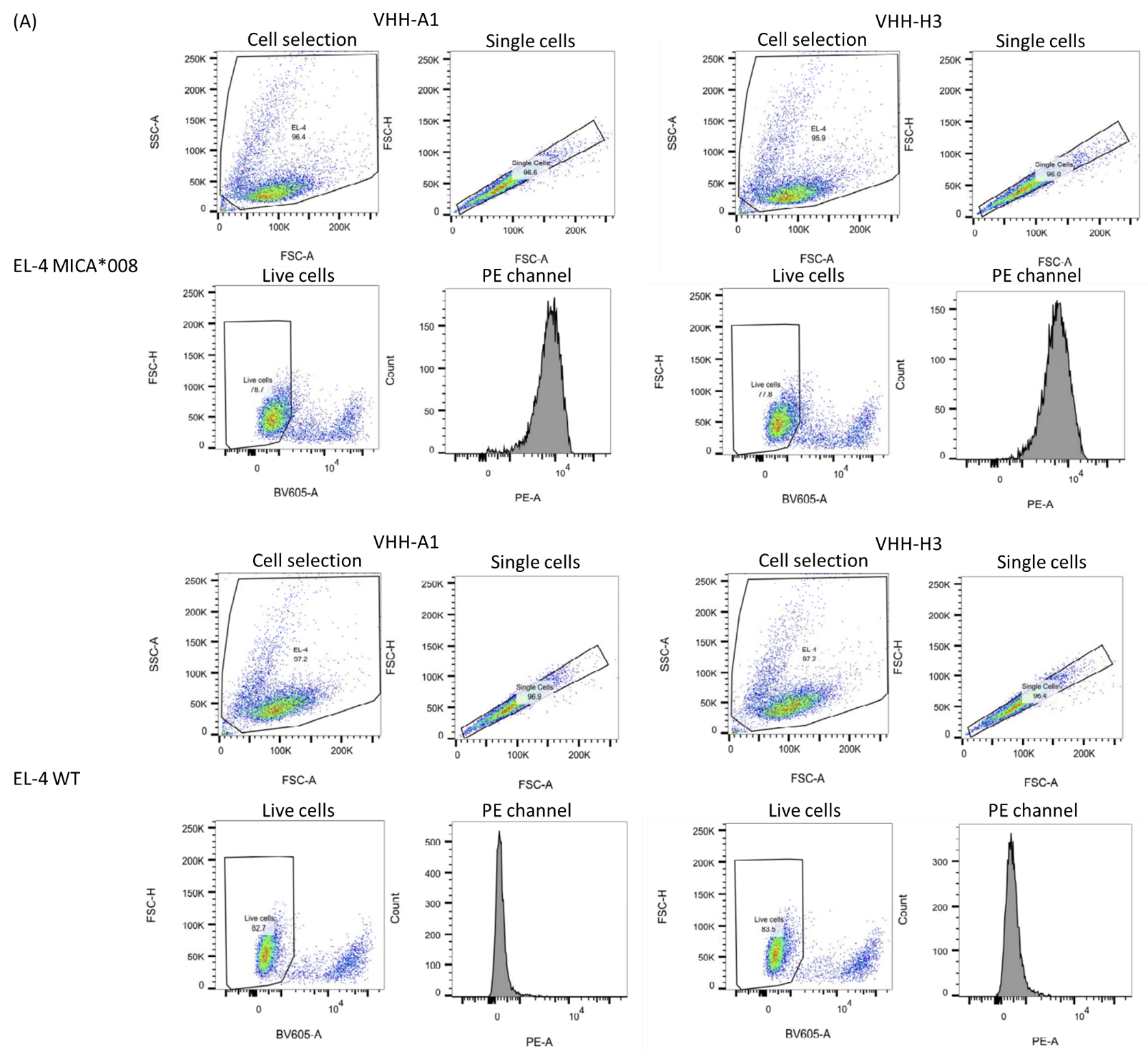
**

**
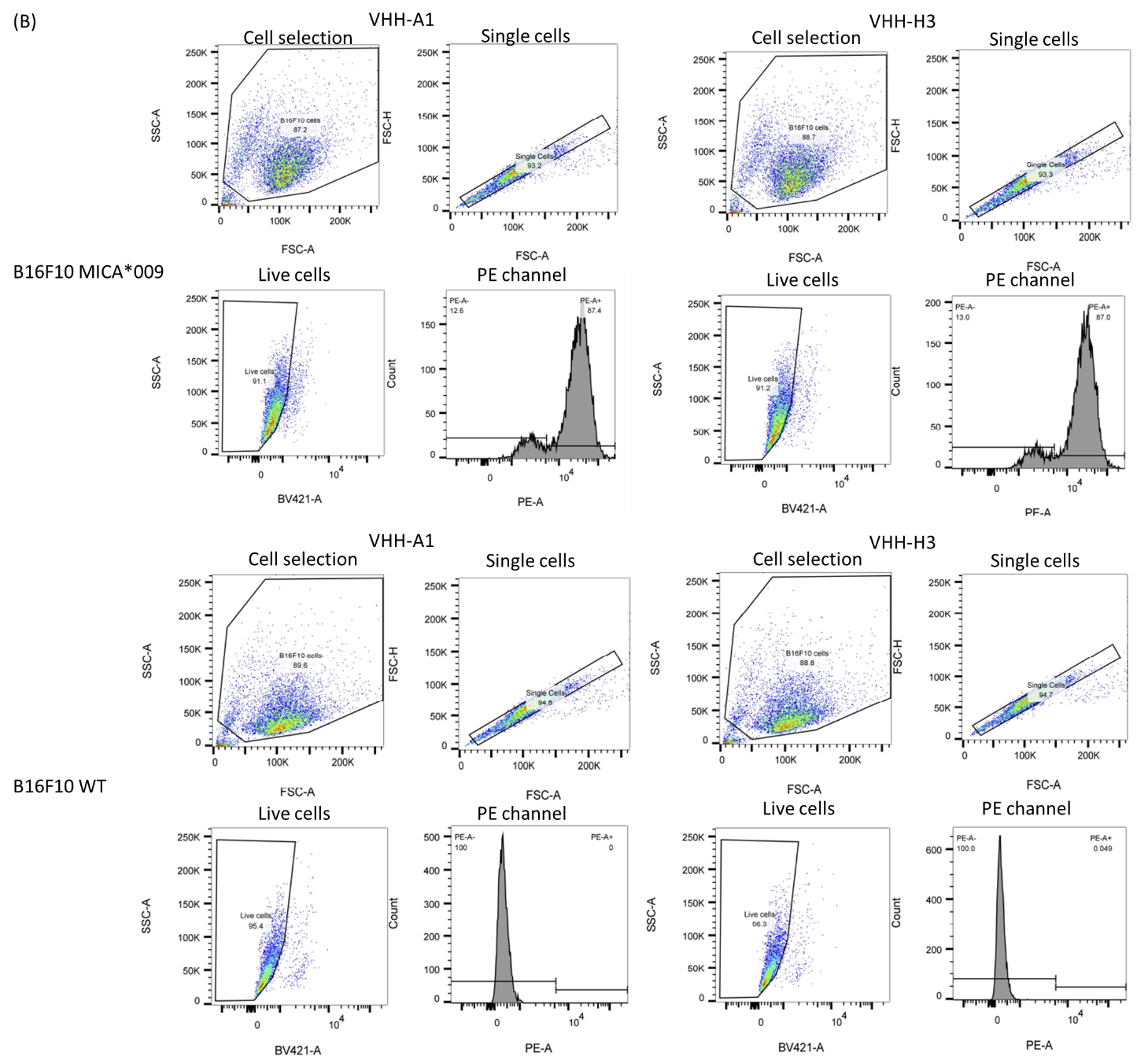
Supplementary figure 4** **Gating strategy to determine VHH-A1 and VHH-H3 binding to surface-disposed MICA on EL-4 (A) and B16F10 (B) cells.** Cells were stained with biotinylated VHH-A1 and VHH-H3 (1 μg/mL) for 30 minutes on ice, washed, and stained with a cocktail of streptavidin-PE (2.5 μg/mL) and propidium iodide (for EL-4) or LIVE/DEAD™ Fixable Violet Dead Cell Stain (for B16F10) for 30 minutes on ice. Cells were washed and analyzed on an LSR Fortessa flow cytometer (BD Biosciences). Gating strategies are shown for cells stained with biotinylated VHH, Streptavidin-PE, and viability dye, but the appropriate negative staining controls were added to determine gates. First all cells were selected based on FSC and SSC. Then, we selected singlets based on FSC-A and FSC-H. We determined viability in the BV605 channel for EL-4 and BV421 channel for B16F10 cells. MICA-staining was determined by signal in the PE channel.


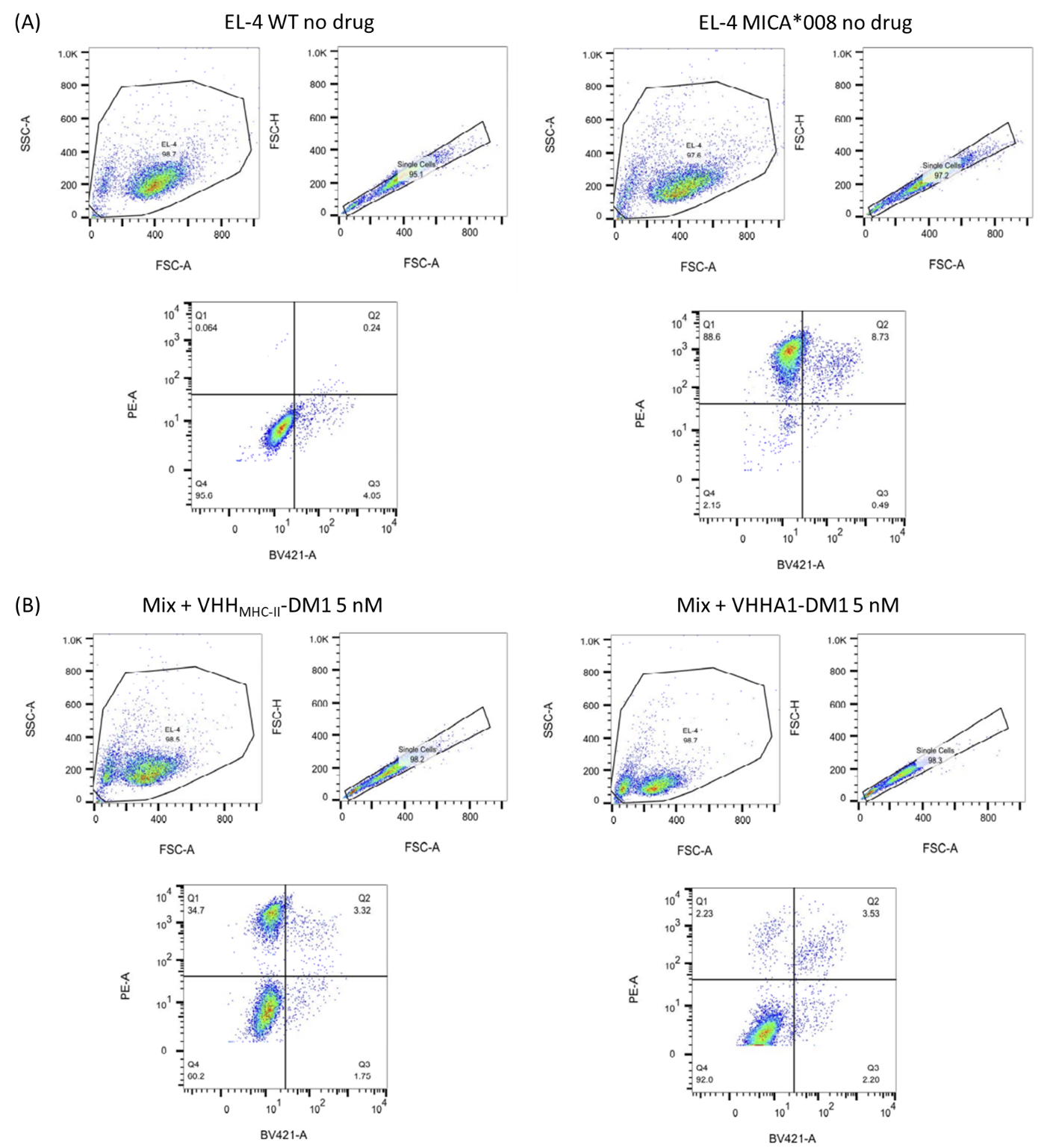
**Supplementary figure 5.** **Gating strategy to determine viability in a mixed population of EL-4 WT and MICA^+^ cells undergoing treatment with nanobody-drug conjugate.** Cells were stained with 0.0006 μg/mL biotinylated anti-human MICA/B antibody (Clone 6D4, Biolegend) for 30 minutes on ice. Cells were washed and incubated with Streptavidin-conjugated PE at 0.0025 μg/mL (Invitrogen) and LIVE/DEAD™ Fixable Violet Dead Cell Stain Kit according to manufacturer’s directions (Invitrogen) for 30 minutes on ice. Viability and MICA positivity were determined by flow cytometry. (A) Gating was performed on unmixed EL-4 WT or MICA^+^ cells which were kept in the same culture conditions, without the addition of drug. Cells were deemed viable if they stained negatively in the BV-421 channel. Cells were deemed MICA^+^ if they stained positive in the PE channel (upper left quadrant) or WT if they stained negative in the PE channel (lower left quadrant). (B) Representative gating pattern for mixed EL-4 WT and MICA^+^ cells, here shown for those treated with 5 nM VHH_MHCII_-DM1 (left panels) or 5 nM VHH_A1_-DM1 (right panels). The ratio of WT and MICA^+^ cells were normalized to the relative ratio of untreated WT:MICA^+^ cells.

| **Target** | **Usage** | **Sequence (5’ -> 3’)** |
| --- | --- | --- |
| **VHH hinge** | Library construction (forward primer) | CTTGCGGCCGCTCAGKTGCAGCTCGTGGAGWCNGGNGG |
| **VHH short hinge** | Library construction (reverse primer) | GATCGGCGCGCCGAGGGGTCTTCGCTGTGGTGCG |
| **VHH long hinge** | Library construction (reverse primer) | GATCGGCGCGCCGGTTGTGCTTTTGGTGTCTTGGG |
| **VHH in phagemid vector** | Forward primer for PCR of VHH sequence from phagemid vector | CGCGGCCCAGCCGGCCATGGCCCAGGTGCAGCTCCAGG |
| **VHH in phagemid vector** | Reverse primer for PCR of VHH sequence from phagemid vector | AGTCCTCCTGAGGAGACGGTGACCTGGGTCCCCTGG |
| **LacZ** | Sequence validation of VHH insertion into pHEN6 vector | CAGGAAACAGCTATGAC |

**Supplementary table 1. Primer sequences for creating the VHH phage library, validation of VHH sequences, and Gibson assembly into the pHEN6 vector.**
